# The Effect of *Mycobacterium avium* subsp. *Paratuberculosis* Infection on the Productivity of Cows in Two Dairy Herds with a Low Seroprevalence of Paratuberculosis

**DOI:** 10.3390/ani10030490

**Published:** 2020-03-15

**Authors:** Agnieszka Wiszniewska-Łaszczych, Katarzyna G. Liedtke, Joanna M. Szteyn, Tomasz Lachowicz

**Affiliations:** 1 Departament of Veterinary Public Health, Faculty of Veterinary Medicine, University of Warmia and Mazury, Oczapowskiego 14, 10-718 Olsztyn, Poland; szteyn@uwm.edu.pl; 2 Voivodship Veterinary Nnspectorate in Olsztyn, Szarych Szeregów 7, 10-072 Olsztyn, Poland; lipkon@poczta.onet.pl; 3 Municipal Polyclinical Hospital in Olsztyn, Niepodległości 44, 10-045 Olsztyn, Poland; lachowicz.tomasz@uwm.edu.pl

**Keywords:** *Mycobacterium paratuberculosis*, milk yield, milk composition, paratuberculosis

## Abstract

**Simple Summary:**

Paratuberculosis is a chronic, progressive enteritis of ruminants, caused by *Mycobacterium avium* subspecies *paratuberculosis*. It affects the productivity of infected dairy cows, causing a reduction in the daily milk yield and basic milk components. The aim of the study was to determine the effect of *Mycobacterium avium* subspecies *paratuberculosis* on the productivity of dairy cows in naturally infected herds with different seroprevalences of paratuberculosis. A decrease in milk yield was observed in cows in herds with a higher seroprevalence. The largest decrease in milk yield and basic milk components was observed in older animals.

**Abstract:**

Paratuberculosis is a chronic, progressive enteritis of ruminants, caused by *Mycobacterium avium* subspecies *paratuberculosis*. It affects the productivity of infected dairy cows, causing a reduction in the daily milk yield and basic milk components. The aim of the study was to determine the effect of *Mycobacterium avium* subspecies *paratuberculosis* on the productivity of dairy cows in two herds. The research materials were serum and milk samples taken from cows from two naturally infected dairy herds. All serum samples were serologically tested using the *Mycobacterium paratuberculosis* Antibody ELISA Kit by IDEXX—Screening and Verification. Seroprevalence differed between the herds (5.7% and 11.3%). Seroprevalence varied also between the groups of lactation. The highest seroprevalence was found in the first lactation group in both herds. The milk yield evaluation and analysis of the basic milk components’ content (protein and fat total solids) were tested once a month during one lactation period. The content of the basic milk components varied depending on the lactation group, as well as the serological status of the cows. A decrease in milk yield was observed in cows in herds with a higher seroprevalence (>11%). The largest decrease in milk yield and basic milk components was observed in older animals (>three lactations).

## 1. Introduction

Paratuberculosis, otherwise referred to as Johne’s disease (JD), is a chronic enteritis in cattle and other ruminants. It is caused by *Mycobacterium avium* subsp. *paratuberculosis* (MAP), an acid-fast bacterium that belongs to the *Mycobacterium avium* complex (MAC). Young calves are the most vulnerable to the infection—the first, asymptomatic signs appear after several months or years [1]. The disease in the herd spreads both horizontally and vertically [2]; it spreads slowly, and the incubation time is long. The clinical symptoms are not very distinctive, and immunosuppression of the animals caused by the MAP infection causes a higher susceptibility to other diseases. [3,4] In a study conducted by Raizmann [5], cows infected with MAP also suffered from other coexisting diseases, such as lameness, pneumonia and mastitis. The types of therapy applied so far have not given the expected results. The prevention and control of the epizootic status of the newly introduced animals is the only effective method of preventing MAP infections. The long disease incubation time, the absence of pathognomonic symptoms, and diagnostic methods characterised by variable sensitivity/specificity hinder correct JD diagnosis [4,6]. The disease appears in cattle herds worldwide. Currently, it is recorded in the majority of European countries [4,7], both of the Americas [5,8,9], Australia and New Zealand [10]. Based on data from 48 countries worldwide, Whittington et al. [11] showed that the prevalence of paratuberculosis is in around 20% of herds, and that in some developed countries, it reaches 40%. Milk obtained from infected animals is a potential source of human infection [12]. MAP has been confirmed in patients with Crohn’s disease, type I diabetes and sarcoidosis. MAP has been recognized as one of the etiological factors of these diseases [13].

The presence of MAP infections in the herd is not only potentially hazardous for human and animal health but can also result in productivity changes [14]. There are many studies indicating economic losses in herds of cows infected with paratuberculosis [7,9,15,16]. They are attributed to an increased risk of premature culling [17,18] and reduced fertility [19]. In addition, paratuberculosis has been associated with reduced milk production in dairy cattle [5,20]. In Poland, no comprehensive studies on the presence of Johne’s disease in dairy cattle herds have been conducted so far. The data from the Chief Veterinary Inspectorate, which maintains records of the officially reported cases, indicate the year by year increases in the number of disease foci. The results of the serological tests of dairy cattle in north-eastern Poland confirm a significantly more frequent occurrence of infections as compared to the reported cases. Seropositive results were recorded more frequently in large dairy cattle herds [21].

There were three primary aims of the study:

(1) To evaluate milk yields from cows with seropositive and seronegative reactions for paratuberculosis.
(2) To analyze the basic components of milk from cows with seropositive and seronegative reactions for paratuberculosis.
(3) To determine whether the number of completed lactations has an impact on productivity and the content of the basic ingredients of milk in seropositive and seronegative animals.

## 2. Material and Methods

### 2.1. Materials

The study was conducted in two commercial dairy herds. These herds are in the evaluation program, which is why we had access to milk yield data for both the whole herd and each cow separately. The length of lactation in individual cows varied from 180 to 345 days. The average length of lactation was 307 days in both herds.

Blood samples of 10 mL in volume were collected from the jugular vein from all cows aged more than 18 months. There were 454 samples from herd A and 424 from herd B. After transport to the laboratory, the blood samples were kept for approx. 24 h until the clots were obtained; next, the serum was transferred to sterile tubes. The prepared serum samples were analyzed by serological tests.

Udder milk samples with a volume of 250 mL were taken from each cow and placed into sterile plastic bottles once a month for one lactation period (~11 months). The samples were transported to the accredited laboratory of the Polish Federation of Cattle Breeders and Dairy Farmers (PFHBiPM).

### 2.2. Serological Tests

All serum samples were serologically tested using the Mycobacterium paratuberculosis Antibody ELISA Test Kit (IDEXX Laboratories, Westbrook, ME, USA). The absorbance of the samples was measured at 450 nm. The presence or absence of the antibody to MAP was determined by the Sample to Positive (S/P) ratio. Those with an S/P ≥ 0.30 were classified as positive, those with 0.15 ˂ S/P ˃ 0.30 were classified as doubtful and those with S/P ≤ 0.15 were classified as negative. Blood samples originating from cows that gave a doubtful test result (10 samples) were verified using the IDEXX Paratuberculosis Verification Ab Test (IDEXX Laboratories, Westbrook, ME, USA). Animals from which samples had a positive result in the verification test were considered to be seropositive. Both tests were conducted according to the manufacturer’s instructions.

### 2.3. Yield and Milk Composition Tests

Milk yield evaluation was conducted based on the analysis of the results of sample milking tests according to the AT4 method (PFHBiPM methods http://www.pfhb.pl/index.php/ocena/metody-oceny). The method is based on data obtained from 11 milking tests during the year, in which the amount of milk produced by each cow is measured (to the nearest 0.1 kg) and representative samples are taken to determine the values of milk parameters, such as fat, protein, and dry weight. The content of milk ingredients was measured in % by weight (g/100 g) with an accuracy of 0.01%. The yield evaluation and the analysis of the basic milk components’ content—protein, fat and total solids—were carried out separately for each cow. The results were analyzed by groups of animals in the first, second, third and subsequent lactation periods, and by animals that showed serologically positive and negative results for MAP.

### 2.4. Statistical Analysis

The statistical analysis was conducted by applying the Statistica 10 PL software package. The t-test for independent samples was used to compare quantitative variables with normal distributions (and homogeneous variances). The Mann–Whitney test was used to compare independent variables on the distribution deviating from the normal and heterogeneous variances.

In examining the relationship between the length of lactation and the average annual yield, the tested herds were statistically analyzed by simple linear regression.

The level of statistical significance was α = 0.05. The results were considered as statistically significant at *p* < 0.05.

## 3. Results

### 3.1. Serological Tests

The results of serological tests are presented in Table 1. Seroprevalence was defined at the level of 5.7% for herd A and 11.3% for herd B. The highest percentage of positive results was found in the group of cows in the third lactation in herd B and in the group of cows in the second lactation in herd A. The highest seroprevalence for JD was found in the group of cows in the first lactation in both herds. The statistical analysis carried out with the χ^2^ test (chi-squared test) showed statistically significant differences, at the assumed significance level of *p* ≤ 0.05, between the number of cows showing seropositive reactions in herds A and B (*p* = 0.003). By analyzing individual periods of lactation, statistically significant differences in the number of seropositive cows were recorded in the third lactation (*p* = 0.047).

### 3.2. Yield and Milk Composition Tests

The statistical analysis of the average milk yield from seropositive and seronegative cows in herd A was carried out using the Mann–Whitney test (data were non-normally distributed). In herd B, the t- test was used because the data were normally distributed. The average milk yield in both herds was high, at 9615 kg of milk/year in herd A and 9766 kg/year in herd B. The seropositive animals in herd A showed a higher average yield, producing 0.725 kg of milk/day more than the seronegative animals. In herd B, on the other hand, the average daily lactation yield of seropositive animals was lower by 0.601 kg of milk/day than that of seronegative animals. These results showed statistical significance at the assumed level of *p* ≤ 0.05 (*p* = 0.014, *p* = 0.010, respectively).

Upon comparing the yields from the seropositive and seronegative animals in the lactation groups, no clear trends were recorded (Table 2).

The content of the basic milk components differed in the individual lactation periods, and between seropositive and seronegative animals as well. The statistical analysis of the basic milk components was carried out using the Mann–Whitney test (data were non-normally distributed). Normally distributed data were observed only when comparing the protein content in milk of seropositive cows from herd A (lactation I and II, I and III lactation, I and ˃III lactation); in this case, the t-test was used. Similarly to the yield, no clear trends between seropositive and seronegative animals in the lactation groups were observed (Table 3). A statistically significant difference (*p* = 0,041) was observed only when comparing the average fat content in the milk of seropositive cows from herd B in lactation II and III. Upon comparing the minimum and maximum yield values and the content of individual milk components, we observed that seropositive cows obtained lower maximum values than seronegative cows.

### 3.3. Simple Linear Regression

Linear regression was used in order to estimate the relationship between the length of lactation in all lactation groups, and the average annual yield in the herds A and B. The results of the analysis showed no significant association between the duration of lactation and the average annual yield, with the significance level *p* ≤ 0.05. The significance level for the analyzed factor was greater than expected, which means that the length of lactation is not statistically significant.

## 4. Discussion

Paratuberculosis is a disease that is difficult to diagnose. Diagnosis is difficult due to the long incubation period, the often-subclinical manifestation of the disease and the non-specific clinical symptoms, as well as diagnostic variability. Choosing the right method to diagnose infection in a herd is the main challenge. The sensitivity of diagnostic tests can vary widely: of serum ELISA from 7% to 94% [22], of fecal culture from 20% to 74% [22,23,24] and of PCR from 4% to 100% [25]. Despite this variability in sensitivity, serological tests are relatively quick and low-cost, and allow the testing of individual animals, as well as herds.

In our studies, we showed a significantly different seroprevalence of paratuberculosis in both herds (5.7% and 11.3%). Similar differences in the seroprevalence of paratuberculosis in cattle herds were also observed by other researchers. In Canada, the seroprevalence in herds ranges from 1.8% to 3.9% [26]; in New Zealand, estimates are around 50–70% [15]; and in some regions of the USA, it may even reach 90% [27]. Based on the collected data, Whittington et al. [11] determined the global prevalence to be 20%.

The milk yield of cows depends on many factors: genetic, physiological and environmental, as well as on health [28]. Thus, MAP infection, like other disease factors, can affect animal productivity. It is therefore worth looking at differences in dairy cow productivity in infected and MAP-free herds. However, this is not easy in the case of Polish cattle herds. We do not have a recognized situation of MAP infection and spread in Poland. Only a few seroprevalence research results are available, pertaining only to the north-eastern part of the country [21,29,30]. In addition, the structure and productivity of dairy herds in Poland are very diverse. Studies by the PFHBiPM showed that the average yield in Poland was 6536 kg/year [31]. It is hard to evaluate the changes in productivity by comparing the average yields in the studied herds, as they are high (over 9000 kg/year) compared to the average milk yields in Poland. Therefore, the effect of MAP infection on milk yield can only be assessed by comparing the yields of seropositive and seronegative animals kept in the same herd. In our study, we observed a statistically significant decrease in the milk yield of seropositive animals in herd B (higher seroprevalence). A similar relationship was observed by Pritchard et al. [6] in the UK and by Bates et al. [15] in New Zealand. Conversely, in herd A (lower seroprevalence), we observed a significantly higher milk yield in seropositive than in seronegative cows. A higher milk yield of seropositive cows was also observed by Johnson et al. [32]. The results published by Benedictus et al. [17], Hendrick et al. [18] and Lombard et al. [14] do not show any differences in milk yield between seropositive and seronegative cows. Some researchers observed differences in the milk yields of seropositive and seronegative cows in individual lactations. Nordlund et al. [33] showed that the statistically significant losses are noticeable only in animals above the fourth lactation. Gonda et al. [34] expressed a different opinion. They suggested that the highest differences in yields are observed between the first and second lactations—the same relationship we observed in our study in herd B. In herd A, we did not observe a relationship between milk yields in individual lactations.

In the herds tested, the cows showed different lengths of lactation. The length of lactation in individual cows varied from 180 to 345 days. However, a simple regression analysis showed no relationship between the length of lactation, the immune status of cows against paratuberculosis, and milk yield in the herd, both in herd A with the lower seroprevalence, and in herd B with the higher seroprevalence. In our opinion, this is due to the large differences in abundance between the populations of seropositive and seronegative cows.

In the presented work, the content of the individual milk components was evaluated. Differences were found in the content of basic milk components during individual lactation periods, and between seropositive and seronegative animals. However, no clear trends were observed. The decrease in the fat and protein content in seropositive cows observed in herd A in the current study was also observed in studies by Vidić et al. [35], Bedenictus et al. [17] and Sweeney et al. [36]. In contrast, the milk from seropositive cows in studies conducted by Lombard et al. [27] and Johnson et al. [32] showed higher protein and fat content compared to that from seronegative cows. Similarly, we showed a slightly higher protein and fat content in the milk of seropositive cows in herd B; however, our results show no statistical significance. Research by Donata et al. [37], on the other hand, indicated that it is possible to decrease the percentage of protein in the milk of MAP-infected animals, without differences in the milk fat content of seropositive and seronegative animals. Upon comparing the minimum and maximum performance values and the content of the individual components of milk from seropositive and seronegative animals, we observed that cows infected with MAP presented maximum values which were much lower than the maximum values obtained from animals which were not infected, in both herds.

Johnson et al. [32] claimed that the presence of antibodies can cause both a decrease and increase in yield, as well as various changes in the milk composition. He suggests that this may be due to different stages of the disease in individual animals, as well as genetic conditions and the efficiency of the immune system. Based on our results, it seems that changes in milk yield depend on the level of seroprevalence in a given herd. In the herd with a lower prevalence (5.7%), an increase in milk yield was observed in seropositive animals, while the milk yield decreased in the herd with a higher seroprevalence (11.3%). Similar conclusions were formulated by Donat et al. [37], stating that the decrease in milk yield, as well as in the percentage content of protein, is related to the within-herd prevalence (WHP).

## 5. Conclusions

Changes in milk yield between seropositive and seronegative MAP cows were identified in both herds. A decrease in milk yield was observed in cows in herds with a higher seroprevalence (>11%). No statistically significant differences in the content of the basic components of milk between seropositive and seronegative animals were observed. The highest decrease in milk yield and the basic components of milk was observed in older animals (above the third lactation). The basic argument for the owner of the herd to implement paratuberculosis control programs in a dairy cattle herd is the impact of the disease on the economic results of the farm. Despite the observed lack of decrease in milk yield in cows from the herd with a low seroprevalence, it should be remembered that a failure to control will result in the spread of infection in the herd, and thus an increase in seroprevalence. Consequently, this will lead to a decrease in milk yield. The costs associated with the presence of infection in the herd would also include the costs of tests and the earlier removal of infected animals from the herd.

## Figures and Tables

**Table 1:** The paratuberculosis seroprevalence in dairy cattle herds divided into groups of lactation.

Result Test								
	Lactation	Screening Test				Verification Test		
		Number of Samples Examined	+	+/−	−	Number of Samples Examined	+	−
Herd A	I	213	9	0	204	0	0	0
	II	136	9	1	126	1	1	0
	III	48 ^a^	3	0	45	0	0	0
	>III	57	4	0	53	0	0	0
	Total	454 ^b^	25	1	428	1	1	0
Herd B	I	139	10	3	126	3	2	1
	II	119	10	3	106	3	2	1
	III	98 ^a^	14	1	83	1	1	0
	>III	68	8	2	58	2	1	1
	Total	424 ^b^	42	9	373	9	6	3

^+^ seropositive; − seronegative; ^+^/− doubtful; statistical significance between results: ^a^ *p* = 0.047; ^b^ *p* = 0.003; no statistical significance was observed between the other results.

**Table 2:** A comparison of the results of the milk yields (kg/day) of seropositive and seronegative cows in two tested herds (A and B).

ELISA Result	Herd A				Herd B			
	+		−		+		−	
	Average (kg/Day)	Min/Max (kg/Day)	Average (kg/Day)	Min/Max (kg/Day)	Average (kg/Day)	Min/Max (kg/Day)	Average (kg/Day)	Min/Max (kg/Day)
I lactation	31.93	10.30/39.60	32.00 ^a^	4.80/51.80	30.23 ^d^	17.90/41.30	32.93 ^d^	7.20/50.10
II lactation	28.45 ^ab^	8.50/53.50	29.9	3.1/60.00	37.99	14.70/55.00	31.83	8.30/67.70
III lactation	38.08	21.70/52.20	31.92 ^b^	4.60/67.10	28.33	4.00/52.90	30.37	0.80/66.40
>III lactation	28.02	11.10/51.30	29.76	5.80/59.10	27.33	6.30/56.60	30.77	6.00/64.40
Whole herd	31.62 ^c^	8.50/53.50	30.89 ^c^	4.60/67.10	30.97 ^e^	4.00/56.60	31.47 ^e^	0.80/67.70

+ seropositive; - seronegative; statistical significance between results: ^a^ *p* = 0.024; ^b^ *p* = 0.011; ^c^ *p* = 0.014; ^d^ *p* = 0.008; ^e^ *p* = 0.010; no statistical significance was observed between the other results.

**Table 3:** A comparison of the percentages (%) of milk components in samples obtained from seropositive and seronegative animals in two tested herds (A and B).

Lactation	ELISA Result	Protein				Fat				Total Solid			
		(% in Milk Content)				(% in Milk Content)				(% in Milk Content)			
		Herd A		Herd B		Herd A		Herd B		Herd A		Herd B	
		Average	Min/Max	Average	Min/Max	Average	Min/Max	Average	Min/Max	Average	Min/Max	Average	Min/Max
I	+	3.15	2.64/3.75	3.14	2.54/4.25	4.08	3.04/6.28	4.09	3.33/7.28	12.73	11.19/15.26	12.71	11.73/15.41
	-	3.19	2.26/5.06	3.07	2.43/4.87	3.99	1.88/8.65	3.82	1.79/7.49	12.69	9.93/18.59	12.47	10.33/17.27
II	+	3.13	2.68/4.36	2.9	2.44/4.66	3.83	2.84/6.07	3.83 ^a^	2.50/6.72	12.99	11.06/15.28	12	10.52/15.71
	-	3.23	2.30/5.24	3.03	2.35/4.99	3.87	1.69/8.80	3.87	1.83/9.00	12.94	10.17/18.56	12.37	10.16/17.36
III	+	3.19	2.76/4.01	3.18	2.54/4.50	3.63	2.76/5.88	3.63 ^a^	1.38/6.52	12.32	11.27/14.26	12.1	10.20/15.80
	-	3.3	2.57/4.77	3.09	2.15/5.27	3.88	1.47/7.73	3.88	1.51/9.00	13.17	9.73/16.50	12.34	9.91/19.60
>III	+	3.28	3.10/4.36	3.2	2.75/4.16	4.21	2.43/6.31	4.21	2.53/5.73	12.99	11.08/15.47	12.9	11.14/14.57
	-	3.28	2.60/5.35	3.13	2.20 /4.73	4.03	2.37/8.89	4.03	1.68/7.53	13.07	10.29/17.90	12.6	9.67/16.60
Whole herd	+	3.18	2.64/4.36	3.1	2.44/4.66	3.93	2.43/6.31	3.94	1.38/7.28	12.75	11.08/15.47	12.42	10.20/15.80
	-	3.25	2.30/5.35	3.08	2.15/5.27	3.94	1.47/8.89	3.9	1.51/9.00	12.96	9.73/18.59	12.44	9.91/19.60

+ seropositive; - seronegative; statistical significance between results: ^a^ *p* = 0,041; no statistical significance was observed between the other results.
